# A guidebook for DISCO tissue clearing

**DOI:** 10.15252/msb.20209807

**Published:** 2021-03-26

**Authors:** Muge Molbay, Zeynep Ilgin Kolabas, Mihail Ivilinov Todorov, Tzu‐Lun Ohn, Ali Ertürk

**Affiliations:** ^1^ Institute for Tissue Engineering and Regenerative Medicine (iTERM) Helmholtz Center Neuherberg, Munich Germany; ^2^ Institute for Stroke and Dementia Research Klinikum der Universität München Ludwig‐Maximilians‐University Munich Munich Germany; ^3^ Munich Medical Research School (MMRS) Munich Germany; ^4^ Graduate School for Systemic Neurosciences (GSN) Munich Germany; ^5^ Munich Cluster for Systems Neurology (SyNergy) Munich Germany

**Keywords:** 3D imaging, DISCO, labeling, light sheet, tissue clearing, Development & Differentiation, Methods & Resources, Neuroscience

## Abstract

Histological analysis of biological tissues by mechanical sectioning is significantly time‐consuming and error‐prone due to loss of important information during sample slicing. In the recent years, the development of tissue clearing methods overcame several of these limitations and allowed exploring intact biological specimens by rendering tissues transparent and subsequently imaging them by laser scanning fluorescence microscopy. In this review, we provide a guide for scientists who would like to perform a clearing protocol from scratch without any prior knowledge, with an emphasis on DISCO clearing protocols, which have been widely used not only due to their robustness, but also owing to their relatively straightforward application. We discuss diverse tissue‐clearing options and propose solutions for several possible pitfalls. Moreover, after surveying more than 30 researchers that employ tissue clearing techniques in their laboratories, we compiled the most frequently encountered issues and propose solutions. Overall, this review offers an informative and detailed guide through the growing literature of tissue clearing and can help with finding the easiest way for hands‐on implementation.

## Graphical abstract

Tissue‐clearing methods have allowed imaging intact biological specimens and understanding biology at a whole‐organism and systems‐level. This Review provides a guide for scientists who would like to setup a clearing protocol, with an emphasis on DISCO methods.

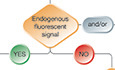

## Tissue transparency

Biological tissues are remarkably complex. While several histological techniques are currently available for the analysis of biological tissues, a variety of challenges remains to be tackled. For example, imaging large samples requires destroying tissue integrity by sectioning them into thin slices for visualization. This process is labor‐intensive and re‐assembling information from serial tissue sections into a three‐dimensional reconstruction is an extremely time‐consuming process, likely causing loss of important information (Ertürk *et al*, 2012a). Rendering the tissues transparent is an option for circumventing the need for sectioning and allows imaging tissues while keeping them intact and preserving their details. Optical tissue clearing is the term that spans a variety of methods applying this concept. From the very first clearing protocol to the most recent ones, transparency is achieved by homogenizing the refractive index (RI) within the tissue. When light waves propagate through a tissue with nonhomogeneous refractive indices, the differences in the RIs will result in refraction at the interface, distorting the shape of the wave front. This process is also referred to as scattering. Having a wider distribution of the RI in a medium causes more scattering, degrading the quality of the acquired image. Therefore, minimizing the refraction by homogenizing the RI allows light to penetrate deeper into the medium (Tuchin, 2015), hence rendering a sample transparent.

**Figure 1:**
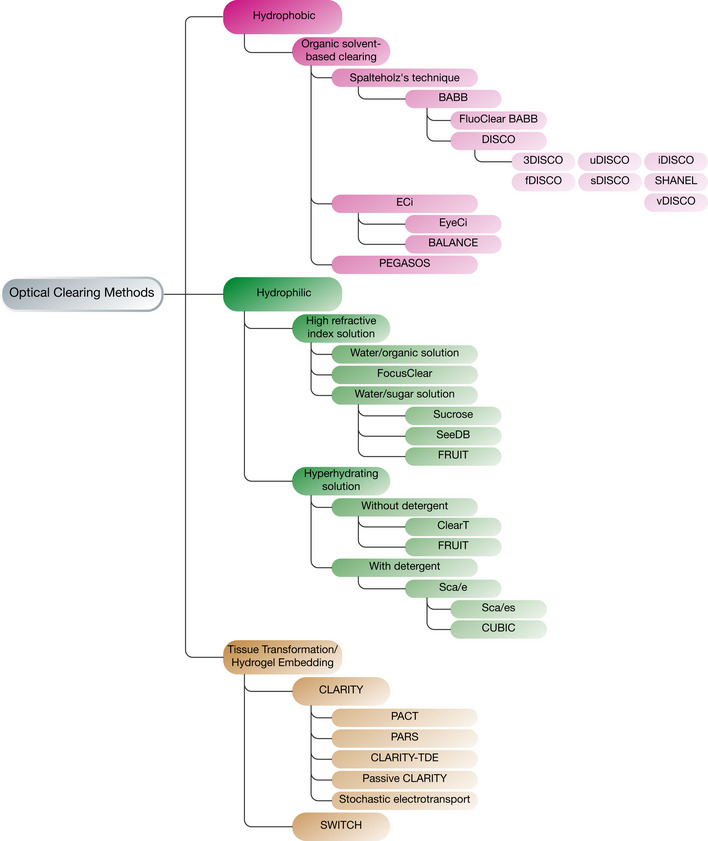
The most common tissue clearing methods Tissue clearing methods can be separated into three main categories based on their chemistry: hydrogel‐based, hydrophilic, and hydrophobic. The subtypes mainly differ according to reagents/approaches used. Several parameters will influence the choice of the procedure such as the labeling method, the size of the tissue, the species, and the nature of the molecule of interest.

The first tissue clearing method was described in the 1900s by Werner Spalteholz, who used a mixture of Methyl salicylate/Benzyl benzoate (5:3 vol:vol) (Spalteholz, 1914) to apply the RI homogenization protocol. Derivatives of the Spalteholz’ method were used by embryologists by substituting methyl salicylate with benzyl alcohol. This new combination formed Murray’s clear (BABB) containing 1:2 combination of benzyl alcohol:benzyl benzoate (Dent *et al*, 1989; Klymkowsky & Hanken, 1991). Currently, tissue clearing, especially when paired with optical imaging and fluorescent labeling of cellular structures has become a powerful tool for collecting information deep within tissues at single‐cell resolution. Due to the wide applicability of tissue clearing, several different approaches have emerged including hydrogel‐based clearing, aqueous‐based clearing, and those that followed Spalteholz's historical recipe on tissue dehydration—solvent‐based clearing (Fig 1) (Silvestri *et al*, 2016). These approaches have expanded the application of tissue clearing by providing specialized protocols for a variety of purposes.

Cleared lipid‐extracted acryl‐hybridized rigid immunostaining/*in situ* hybridization‐compatible tissue hydrogel (CLARITY) (Chung *et al*, 2013), passive CLARITY technique (PACT) and perfusion‐assisted agent‐release *in situ* (PARS) (Yang *et al*, 2014) are hydrogel‐based protocols. They utilize an acrylamide monomer to form a tissue‐hydrogel hybrid, which allows immobilizing most of the amino group containing molecules by cross‐linking, thus keeping large tissues intact and decreasing structural damage. This process involves electrophoresis or diffusion depending on the nature of the biological specimen and the specific scientific question. After fixing the tissue in a hydrogel scaffold, the sample is subjected to delipidation using sodium dodecyl sulfate (SDS) detergent. The composition of the reagents can be modified depending on the intended purpose of imaging (e.g., RNA or protein imaging). The nature of the reagents also facilitates tissue expansion which potentially reveals hidden structures. Among the downsides of this approach are the longer incubation times required for clearing and the need for special equipment in order to employ some of the techniques (Gradinaru *et al*, 2018). All subtypes derived from the CLARITY approach such as PACT and PARS (Yang *et al*, 2014), stabilization under harsh conditions via intramolecular epoxide linkages to prevent degradation (SHIELD) (Park *et al*, 2019) and BoneCLARITY (Greenbaum *et al*, 2017) are protocols optimized toward a certain use case. As an example, PACT is a passive CLARITY technique involving a protocol suitable for small samples, while PARS is a perfusion‐assisted agent‐release *in situ* protocol for whole‐body clearing with solution delivery through the vasculature. Other CLARITY‐based protocols are modified for different applications or specific tissues, such as BoneCLARITY, developed for investigating the notoriously difficult bone tissue. An elaborate review on hydrogel‐based tissue clearing methods is available by Gradinaru *et al* (2018).

The second major type of tissue clearing methods is hydrophilic or aqueous‐based approaches. These methodologies initially surfaced around the 1990s utilizing various water‐soluble agents such as sugars, dextran, sucrose, urea, and amino alcohols. The main distinctive feature of aqueous‐based methods is that the employed water‐soluble agents are less destructive to the tissue and display high levels of biocompatibility and biosafety. The different method subtypes stem from the different reagents that are used in protocols for decolorization, delipidation, or RI‐matching steps, i.e., urea in Sca/e (Hama *et al*, 2011), urea with sorbitol in Sca/eS (Hama *et al*, 2015), fructose for See Deep Brain (SeeDB) (Ke *et al*, 2013), and amino alcohols in clear, unobstructed brain or body imaging cocktails and computational analysis (CUBIC) (Tainaka *et al*, 2014). Hydrophilic methods involve several alternative mechanistic approaches for homogenizing the RI. For instance, the sample can be passively immersed in an RI‐matching solution. Alternatively, lipids can be removed from the sample and tissues can be hyperhydrated to lower the RI. For more details on hydrophilic approaches, an extensive review is available by Tainaka *et al* (2018).

Lastly, hydrophobic or solvent‐based clearing methods rely on organic solvents to render the tissues transparent. While this approach dates back to Spalteholz’ protocol described in 1911, it did not draw much attention at the time with the exception of a few publications elaborating on the method (Dent *et al*, 1989; Klymkowsky & Hanken, 1991). It later re‐emerged with 3D imaging of solvent‐cleared organs (3DISCO) that was used to achieve transparency of a whole mouse brain in 1–2 days (Ertürk *et al*, 2012a). Every hydrophobic clearing method involves three fundamental steps: dehydration, delipidation and RI matching. The dehydration step is the distinctive step for hydrophobic methods and together with delipidation, the aim is to remove the two most abundant chemical components of biological tissue, namely water and lipids. The RI of water is 1.33 (Hale & Querry, 1973) and greatly differs from that of the remaining tissue (~ 1.55). Therefore, replacing water with a liquid with a similar RI to that of the remaining tissue can greatly reduce scattering (Richardson & Lichtman, 2015).

## Applications of tissue clearing

Tissue clearing offers the opportunity to understand biology at a whole organism and systems level and presents several advantages for making new biological discoveries. One major feature of tissue clearing is its potential to reveal 3‐dimensional structural information. The ability to keep tissues intact during imaging has revealed previously unknown microscopic details and anatomical connections. For instance, imaging of cleared bone marrow using 3DISCO showed that non‐dividing stem cells localize in perisinusoidal areas in the bone marrow (Acar *et al*, 2015). Another study used FocusCLEAR to reveal the spatial organization of cockroach brain nuclei (Chiang *et al*, 2001). In the mouse gut, immunolabeling‐enabled imaging of solvent‐cleared organs (iDISCO) revealed the peripheral nervous system adapting to perturbations (Gabanyi *et al*, 2016). New cortical brain regions, which are downstream of whisker‐evoked sensory processing, were discovered using iDISCO+ in mice (Renier *et al*, 2016). More recently, imaging of whole‐body neuronal projections in adult mice using vDISCO (the ‘v’ refers to the variable domain of heavy‐chain antibodies; that is, nanobodies) revealed vascular connections between the skull and the meninges (Cai *et al*, 2019), similar to the discovery of trans‐cortical vessels in the long bones using the simpleCLEAR protocol (Grüneboom *et al*, 2019). Additionally, clearing agent fructose, urea, and glycerol for imaging (FUnGI) provided an organoid clearing protocol for multi‐color lineage tracing of tumor heterogeneity (Rios *et al*, 2019).

**Figure 2:**
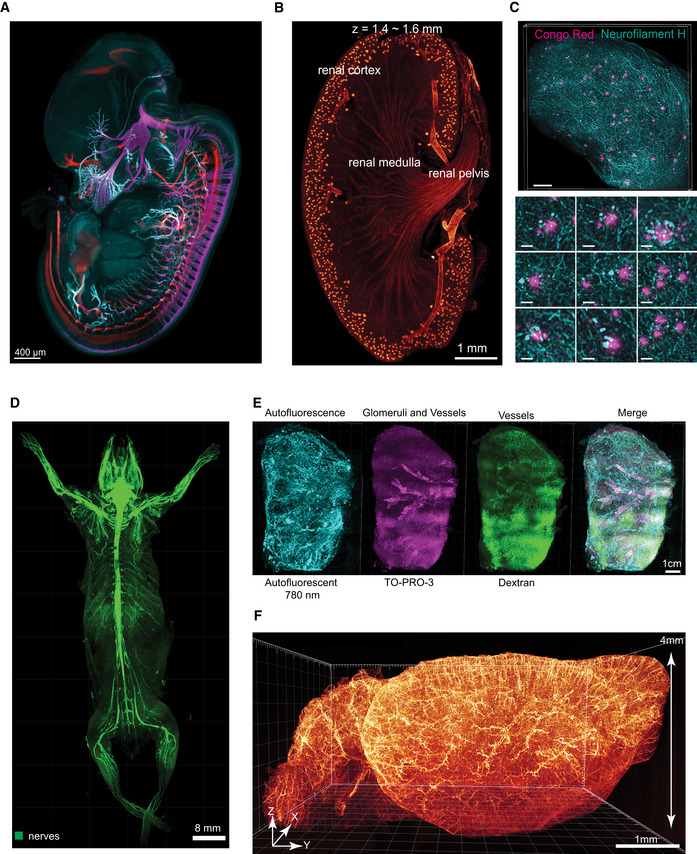
Tissue clearing can be applied to samples ranging from organs, to mouse embryos to adult rodent bodies and reveals macroscopic and microscopic details (A)Nerve and nerve endings are revealed in an E12.5 mouse embryo cleared with iDISCO protocol and immunostained for motor (red) and sensory (magenta) nerves and nerve endings (cyan).(B)Kidney vasculature is visualized after fDISCO clearing using CD31‐A647 labeling.(C)B‐amyloid plaques in the brain with neurofilament H staining in a 10‐month‐old in AD mouse cortex cleared with iDISCO (Scale bar: 200 μm in upper and 30 μm in lower figures).(D)Neuronal projections in the whole adult mouse are shown in a Thy1‐GFPM mouse boosted with Anti‐eGFP nanobodies using the vDISCO clearing protocol.(E)A 3D reconstruction of whole adult human kidney performed using SHANEL. The autofluorescence signal at 780 nm (cyan), the glomeruli and vessels from TO‐PRO‐3 labeling (magenta), the vessels from the dextran labeling (green), and the merged image are shown, respectively.(F)Whole‐brain vasculature is shown using PEGASOS protocol in Tie2‐Cre;tTAflox;tetO‐H2BGFP (TTH) mice in lateral view. Used with permission from: (A) Dr. Gist Croft, Rockefeller University, (B) *Science Advances* (Qi *et al*, 2019), (C) *Cell* (Liebmann *et al*, 2016), (D) *Nature Neuroscience* (Cai *et al*, 2019), (E) *Cell* (Zhao *et al*, 2020), (F) *Cell Research* (Jing *et al*, 2018).

Besides revealing detailed information, keeping the tissues intact also maximizes the information that can be obtained from a large specimen. Elegant examples of this are whole‐body analyses that allowed the unbiased assessment of treatment efficacy and enabled analyzing the biodistribution of nanoparticles, antibodies, and various other targeting agents. This powerful application was demonstrated with the use of CUBIC to detect cancer metastases throughout a whole mouse body sample (Kubota *et al*, 2017). More recently, vDISCO, in conjunction with deep learning‐based algorithms allowed the quantification of cancer cells that had been targeted by therapeutic antibodies (Pan *et al*, 2019). Beyond cancer research, further medically relevant applications that are worth noting include the analysis of the spatial distribution of transplanted stem cells in adult mice body using ultimate DISCO (uDISCO) (Pan *et al*, 2016) and screening the transfection efficiency of adeno‐associated virus (AAV) variant with CLARITY (Deverman *et al*, 2016). Furthermore, iDISCO+ enabled automated cell detection of cFos staining and Allen Brain Atlas registration revealing the brainstem circuit control for feeding behavior and B‐amyloid plaques in an Alzheimer’s disease (AD) mouse model (Liebmann *et al*, 2016; Nectow *et al*, 2017) (Fig 2).

**Figure 3:**
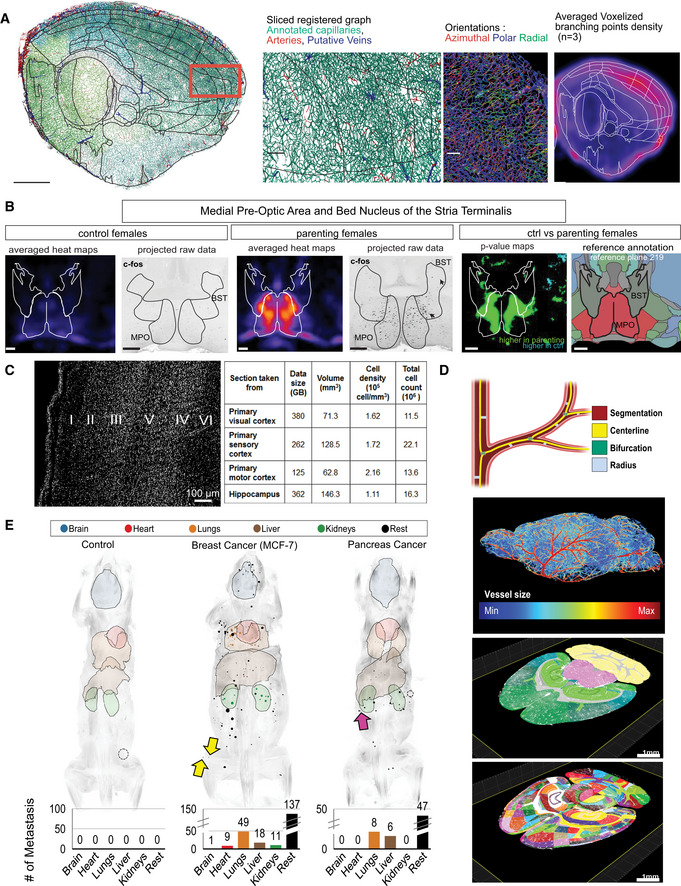
Analysis tools for tissue clearing applications Analysis tools include: 
(A)TubeMap pipeline which demonstrates the fine‐scale organization on the brain vasculature. In this study, Kirst *et al* (2020) demonstrated how stroke affects the brain, using antibody labeling.(B)The ClearMAP pipeline is used for examining parental behavior through Fos activity in the whole brain followed by a filter‐based analysis.(C)SHANEL pipeline has one of the recent algorithms that include deep learning methods to analyze big tissues to quantify cleared human brain tissues identified in the six layers of primary visual cortex. The summary of the cell properties from different brain regions taken from cortex and hippocampus were analyzed using the authors’ CNN.(D)The VesSAP pipeline, which can extract features and registers the mouse brain vasculature to Allen Brain Atlas. Images represent the steps of feature extraction, radius illustration, and vascular segmentation.(E)The DeepMACT pipeline, which detects metastasis throughout organs in adult mice. Each dot represents a metastasis. Used with permission from (A) *Cell* (Kirst *et al*, 2020), (B) *Cell* (Renier *et al*, 2016), (C) *Cell* (Zhao *et al*, 2020), (D) *Nature Methods* (Todorov *et al*, 2020), (E) *Cell* (Pan *et al*, 2019).

Another benefit of whole intact tissue visualization is that it provides less error‐prone measurements and more informative quantification. For example, the growth of glioblastomas in mice was measured under different conditions using 3DISCO clearing (Garofalo *et al*, 2015) and the distance between neural stem cells and blood vessels was assessed with Sca/e (Hama *et al*, 2011). Moreover, clearing combined with unbiased quantification methods, i.e., deep learning algorithms, made it possible to obtain a system‐level understanding at single‐cell resolution. The complex mouse brain vasculature was inspected using a combination of 3DISCO and a convolutional neural network and revealed structural differences in the cerebral angioarchitecture among common inbred and outbred mice strains (Todorov *et al*, 2020; Fig 3). The same pipeline, but relying on manual analysis instead of machine learning, would be significantly more time‐consuming and might potentially result in a subjective end result. A similar approach involving iDISCO+ revealed the local adaptations and functional correlates across brain regions and indicated vascular plasticity in diverse disease models (Kirst *et al*, 2020).

The ability of clearing to reveal fine details deep within tissues makes it a powerful tool in the context of disease pathology. Analyses of tumors using CUBIC indicated a heterogenous nature of macrophage infiltration in lung carcinoma (Cuccarese *et al*, 2017), while BABB clearing revealed growth patterns of prostate cancer (van Royen *et al*, 2016). Similarly, tissue clearing has been applied to neurological pathology, where 3DISCO showed microvessel reorganization following stroke (Lugo‐Hernandez *et al*, 2017), and CLARITY elucidated the relationship between monoaminergic fibers and Lewy bodies in Parkinson patients (Liu *et al*, 2016), and AD plaques (Ando *et al*, 2014). CLARITY was also used to examine the dynamics of pancreatic innervations in pathological conditions such as in diabetes (Hsueh *et al*, 2017). Overall, the ability of clearing techniques to reveal information that has been inaccessible to other methodologies has been instrumental in the analysis of several pathologies.

In summary, a variety of clearing techniques is available, with each of them presenting certain advantages depending on various parameters or being better suited for given applications. These methods are undergoing continuous improvements and are being adapted to specific applications. Further applications are shown in Table 1, and two recent reviews provide an in‐depth discussion of the different applications (Ueda *et al*, 2020; Tian *et al*, 2021). In the following section, we narrow down our focus to solvent‐based method by first sharing some of the known protocols, developed for various needs.

**Table 1:** Tissue clearing applications based on tissue clearing method and labeling.

Sample	Tissue clearing method used	Labeling
Whole mouse organ		
Development of heart	CUBIC (Li *et al*, 2016)	AAV, Ab labeling
Lung	3DISCO (Ertürk *et al*, 2012a; Mzinza *et al*, 2018)	Endogenous GFP
	SWITCH (Murray *et al*, 2015)	Endogenous GFP
	CLARITY (Kim *et al*, 2015)	Endogenous GFP
	uDICSO (von Neubeck *et al*, 2018)	Endogenous mCherry
Spleen	3DISCO (Ertürk *et al*, 2012a)	Endogenous GFP
Lymph node	3DISCO (Ertürk *et al*, 2012a),	Ab labeling, Endogenous xFP
	3DISCO (Ertürk *et al*, 2012a)	Ab labeling, Endogenous xFP
Mammary gland	CUBIC (Davis *et al*, 2016)	Endogenous GFP
	FUnGI (Rios *et al*, 2019)	Ab labeling
Heart	CLARITY (Kim *et al*, 2015)	Endogenous GFP
	SWITCH (Murray *et al*, 2015)	Ab labeling
Kidney	CLARITY (Kim *et al*, 2015)	Endogenous GFP
Liver	CLARITY (Kim *et al*, 2015)	Endogenous GFP
	SWITCH (Murray *et al*, 2015)	Endogenous GFP
	3DISCO (Mzinza *et al*, 2018)	Endogenous GFP
Uterus	3DISCO (Yuan *et al*, 2018)	Endogenous GFP
Intestine	iDISCO (Gabanyi *et al*, 2016)	Ab labeling
Human organ parts		
Lung	CUBIC (Tainaka *et al*, 2018; Nojima *et al*, 2017)	Ab labeling
Kidney	CUBIC (Tainaka *et al*, 2018; Nojima *et al*, 2017)	Ab labeling
Pancreas	CLARITY (Hsueh *et al*, 2017)	Ab labeling
Spleen	CUBIC (Nojima *et al*, 2017)	Ab labeling
Intestine	CUBIC (Nojima *et al*, 2017)	Ab labeling
Lymph node	CUBIC (Nojima *et al*, 2017)	Ab labeling
Embryo		
Human	3DISCO/iDISCO (Yuan *et al*, 2018)	Ab labeling
Mouse	3DISCO (Ertürk *et al*, 2012b)	Endogenous mGFP, Tomato
	iDISCO (Renier *et al*, 2014)	Ab labeling
	Sca/e (Hama *et al*, 2011)	Endogenous YFP, Ab labeling
	CUBIC (Cuccarese *et al*, 2017; Li *et al*, 2016)	Endogenous GFP, Ab labeling
Brain organoid	iDISCO (Birey *et al*, 2017)	Ab labeling
Bone		
Long bones	CUBIC (Chen *et al*, 2016, 5)	Endogenous mCherry, Ab labeling
	simpleCLEAR (Grüneboom *et al*, 2019)	Endogenous tdTomato, Ab labeling
	3DISCO (Acar *et al*, 2015)	Endogenous GFP, Ab labeling
Whole‐body	vDISCO (Cai *et al*, 2019)	Endogenous GFP, Nanobooster
	PARS (Yang *et al*, 2014)	Ab labeling
	CUBIC (Tainaka *et al*, 2014; Susaki *et al*, 2015)	Endogenous GFP, Ab labeling
	uDISCO (Pan *et al*, 2016)	Endogenous GFP
	vDISCO (Pan *et al*, 2019)	Endogenous mCherry, Ab labeling
	CUBIC (Kubota *et al*, 2017)	Endogenous mCherry, Ab labeling
Brain		
Whole mouse brain	3DISCO (Ertürk *et al*, 2012a; Ertürk *et al*, 2012b; Lin *et al*, 2018; Lugo‐Hernandez *et al*, 2017)	Endogenous GFP, FITC Dextran
	uDISCO (Pan *et al*, 2016; Li *et al*, 2018)	Endogenous GFP
	CUBIC (Tatsuki *et al*, 2016; Susaki *et al*, 2014; Tainaka *et al*, 2018; Susaki *et al*, 2015)	Ab labeling
	iDISCO (Renier *et al*, 2014)	Ab labeling
	iDISCO (Liebmann *et al*, 2016)	Ab labeling
	iDISCO+ (Renier *et al*, 2016; Nectow *et al*, 2017)	Ab labeling
	CLARITY (Kim *et al*, 2015; Bedbrook *et al*, 2018; Deverman *et al*, 2016; Chung *et al*, 2013)	Endogenous GFP, AAV AAV, Endogenous YFP
	Sca/eS (Hama *et al*, 2015)	Endogenous YFP, Ab labeling
	SWITCH (Murray *et al*, 2015)	Ab labeling
	SeeDB2 (Ke *et al*, 2016)	Endogenous YFP
Human brain slice	SHANEL (Zhao *et al*, 2020)	Ab labeling
	SWITCH (Murray *et al*, 2015)	Ab labeling
	CUBIC (Tainaka *et al*, 2018; Nojima *et al*, 2017)	Ab labeling
	CLARITY (Ando *et al*, 2014; Phillips *et al*, 2016; Morawski *et al*, 2018; Liu *et al*, 2016; Chung *et al*, 2013)	Ab labeling
Cockroach brain	FocusClear (Chiang *et al*, 2001; Liu & Chiang, 2003)	Ab labeling, WGA
Rat brain	FluoClearBabb (Stefaniuk *et al*, 2016)	Ab labeling
Fly brain	SeeDB2G (Ke *et al*, 2016)	Ab labeling
Vasculature		
Mouse whole brain	3DISCO (Todorov *et al*, 2020)	WGA, small dye
	iDISCO+ (Kirst *et al*, 2020)	Ab labeling
Tissue		
Spinal cord injury	3DISCO (Ertürk *et al*, 2012a; Zhu *et al*, 2015)	Endogenous GFP
	CLARITY (Hsueh *et al*, 2017; Glaser *et al*, 2017)	Ab labeling, WGA
Tumor–Human	CUBIC (Nojima *et al*, 2017)	Ab labeling
	BABB (van Royen *et al*, 2016)	Ab labeling
	DIPCO (Tanaka *et al*, 2017)	Ab labeling
	FUnGI (Rios *et al*, 2019)	Ab labeling
Others		
Bio‐artificial skeletal muscle	3DISCO, Clear^T2^, ScaleA2 (Decroix *et al*, 2015)	Endogenous GFP, Ab labeling
Cancer		
Lung carcinoma	CUBIC (Cuccarese *et al*, 2017)	Endogenous GFP, RFP, Ab labeling
	3DISCO (Garofalo *et al*, 2015)	Ab labeling
Glioma	vDISCO (Pan *et al*, 2019)	Endogenous mCherry, Ab labeling
Whole body	CUBIC (Kubota *et al*, 2017)	Endogenous mCherry, Ab labeling

## Organic solvent‐based tissue clearing techniques

Solvent‐based or hydrophobic methods have improved drastically in the past decade. New protocols have emerged either addressing the shortcomings of the original protocol or optimizing it for a more specific application. Dehydration, lipid extraction, and RI matching around 1.55 are the essential steps common to this type of clearing approach. The first method to revive the century‐old technique was 3DISCO, which is known to be robust and to work on a number of different tissue types. The major advantages were the speed, the transparency, and the ease of storing the samples, because of their solid nature following processing. However, at the time the main shortcomings were quenching the genetically expressed fluorescence and tissue shrinkage. Nevertheless, these shortcomings also triggered the further optimization of this approach.

### Spalteholz’s tissue clearing

**Figure 4:**
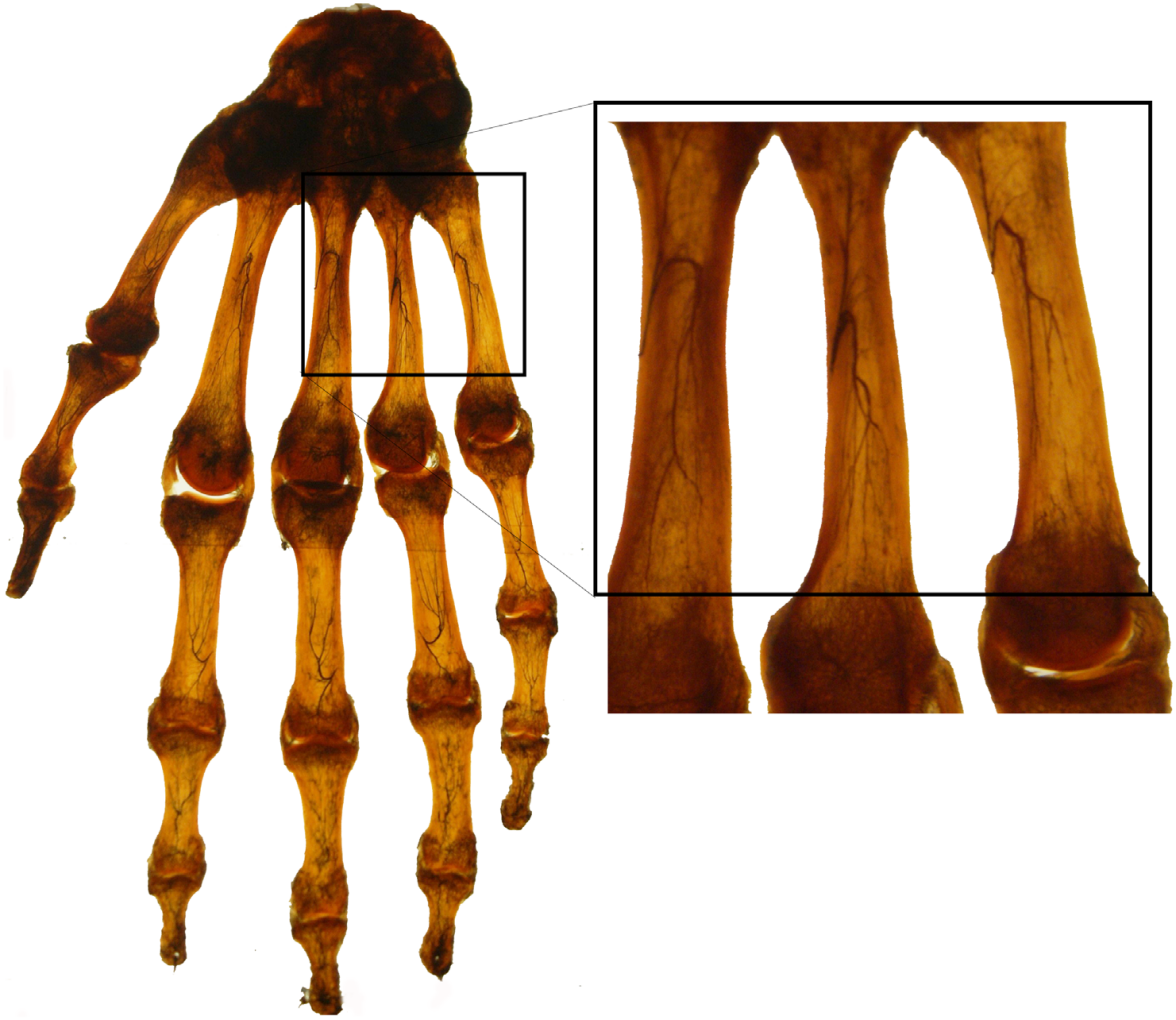
Hand sample cleared by Spalteholz himself in 1911 Spalteholz injected Indian ink to the hand's arteries via the A. nutriciae. Decalcification allowed observing the arterial branching in the bone. The magnified image depicts the inner arterial supply of the metacarpal bones. Originally, this specimen was shown in the 1st exhibition in 1911, “Welthygieneausstellung”, which attracted more than a million people. Most of Spalteholz’s other original specimens were lost during the fire in the Leipzig anatomy department during the 2nd World War. Image: courtesy of Prof. Dr. Hanno Steinke from Anatomy Department of Leipzig University, Germany.

At the beginning of the 20th century, Werner Spalteholz mixed Methyl salicylate and Benzyl benzoate (5:3 vol:vol) to make various human organs transparent (Spalteholz, 1914). Examples of analyzed organs include the human heart which was made transparent in order to observe the blood vessels and the development of an infarction and the human hand which has been preserved to this day in the Anatomy Institute of Leipzig University, Germany (Fig 4). Although Spalteholz’s attempts were very popular in his time, he could only obtain limited scientific knowledge due to the technical limitations of the microscopes.

### BABB clearing

In this protocol, the authors first introduced the term ultramicroscopy which advanced imaging in comparison with the previous techniques via two‐sided light sheets (Dodt *et al*, 2007). They used a series of different ethanol concentrations to dehydrate samples and BABB to clear the tissue. This way, they performed the first imaging of fluorescently labeled embryonic mouse brains and vasculature that was cleared with organic solvents.

### DBE clearing

This protocol substituted the RI‐matching reagent with dibenzyl ether (DBE), a more “GFP‐friendly” clearing medium, alleviating the loss of fluorescence quality caused by BABB (Becker *et al*, 2012). Clearing with DBE provides improved tissue transparency and strikingly improved fluorescence intensity in GFP expressing mouse brains and other samples such as mouse spinal cords, or embryos. In this protocol, ethanol was replaced by tetrahydrofuran (THF) after the realization of ethanol causing the bleaching of endogenous fluorescence. THF was found to be more GFP friendly as a dehydration medium after screening several chemicals.

### 3DISCO

3DISCO was the first protocol that coined the term DISCO, which was then followed by newer iterations (Ertürk *et al*, 2012a, 2012b). Primarily developed for the clearing and imaging of unsectioned mouse brain and spinal cord, the 3DISCO protocol uses THF for dehydration and DCM for lipid extraction. Dibenzyl ether (DBE) is used as the last reagent to match the RI of the sample to the imaging solution.

### iDISCO – iDISCO+

iDISCO (Renier *et al*, 2014) improved the sample labeling conditions by reducing the autofluorescence and increasing the signal‐to‐noise ratio. This protocol allowed antibodies to penetrate deeper into the tissue by the addition of a pretreatment step with methanol, hydrogen peroxide, detergents, and dimethyl sulfoxide (DMSO). This was then followed by antibody labeling and tissue clearing with 3DISCO. In the later iDISCO+ (Renier *et al*, 2016) protocol, an additional delipidation step with DCM was established.

### FluoClearBABB

FluoClearBABB is a derivative of the BABB clearing protocol (Schwarz *et al*, 2015). It differs from the other BABB‐based clearing protocols in that it is performed under basic pH and uses 1‐propanol or *tert*‐butanol for dehydration. FluoClearBABB allows whole‐brain clearing, minimizes optical distortions, and preserves the majority of EGFP and mRFP fluorescence.

### uDISCO

uDISCO was the first technique that accomplished both clearing and imaging an entire mouse body (Pan *et al*, 2016). This was achieved by isotropic shrinkage of the samples by the chemicals. Specifically, the protocol involves immersing the samples into ascending concentrations of tert‐butanol, instead of THF for dehydration and using BABB with diphenyl ether (DPE) for RI matching, which enhances the preservation of endogenous fluorescence.

### ECi clearing

In the ECi protocol, serial dehydration is performed using pH‐adjusted ethanol and the RI matching is done using the non‐toxic, organic compound ethyl‐3‐phenylprop‐2‐enoate (ECi, RI > 1.5) (Klingberg *et al*, 2017). The major advantage of this technique is that it uses non‐toxic reagents for this process.

### vDISCO

The vDISCO protocol includes decolorization, decalcification, permeabilization, and nanoboosting steps in a pressure‐driven pump system (Cai *et al*, 2019). Typically, an active pumping system is known to distort the specimen. However, in this protocol there is very little if any distortion. Still, it is noteworthy that quantification of any tissue distortion have not been reported. Following the pre‐processing steps, a modified 3DISCO protocol is applied through the entire mouse body. Anti‐XFP nanobodies conjugated to fluorescent dyes are used to enhance the fluorescent signal two orders of magnitude and yield single‐cell resolution images from the whole organism.

### PEGASOS

Polyethylene glycol (PEG)‐associated solvent system (PEGASOS) renders nearly all types of tissues transparent except for pigmented epithelium (Jing *et al*, 2018). According to the authors’ report, hard tissues including bones and teeth become nearly invisible after clearing. The PEG component within the clearing medium allows preserving endogenous fluorescence for a long time. In this study, the authors applied a modified uDISCO protocol where they mixed PEG with the reagents.

### FDISCO

FDISCO upgraded the technology with superior fluorescence preserving capability by adjusting the temperature and pH conditions of the 3DISCO protocol (Qi *et al*, 2019). In FDISCO, all steps are performed at 4°C and the pH of all solutions is adjusted to 8.3.

### sDISCO

Stabilized DISCO (sDISCO) provides a solution to optical clearing and storage times for transgenic specimens which are limited due to the continuous formation of peroxides and aldehydes, severely quenching the fluorescence (Hahn *et al*, 2019). It uses purified DBE or BABB and anti‐oxidant propyl gallate is added to stabilize the solutions.

### BALANCE

Bleaching‐Augmented soLvent‐bAsed Non‐toxic ClEaring (BALANCE) is an ECi based clearing protocol that was successfully used to image the mouse heart (Merz *et al*, 2019). The major advantage here is the relatively smaller number of chemicals that are required, ensuring low toxicity. However, since it is a rather new technique it has not been compared with other clearing technologies.

### EyeCi

EyeCi is a combination of the iDISCO and ECi protocols used for immunolabeling and clearing, and has been used on whole eyes of mice (Henning *et al*, 2019). This protocol is specifically adapted for bleaching the melanin in the pigmented epithelium of the retina.

### SHANEL

The Small micelle‐improved Human organ ANtibody Efficient Labeling (SHANEL) protocol was developed to allow the penetration of molecules deep into sturdy aged human tissues and provides a solution to a major bottleneck of human tissue histology (Zhao *et al*, 2020). SHANEL uses a detergent called (3‐((3‐Cholamidopropyl) dimethylammonium)‐1‐propanesulfonate) (CHAPS) for tissue permeabilization. It allows penetration of labeling and clearing agents into centimeters‐thick mammalian organ samples. Human samples two to three orders of magnitude larger than mouse tissues were successfully labeled and cleared by SHANEL.

**Figure 5:**
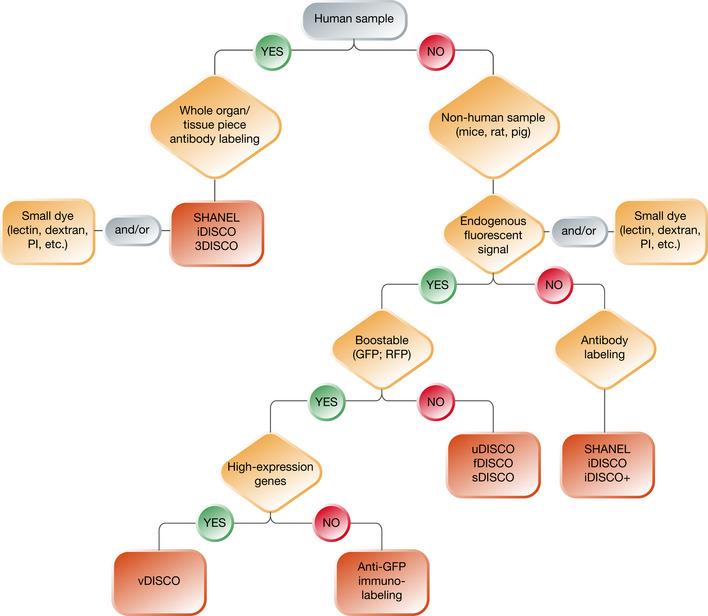
Decision tree for choosing the optimal tissue clearing protocol Several parameters should be weighed to achieve optimal results when choosing the clearing protocol. This flowchart demonstrates an easy way to decide which clearing method would suit a potential research question.

Choosing the best strategy among all the available clearing protocols may require not only expertise but also testing multiple protocols to achieve optimal results. In Fig 5, we offer a simplified chart to help choosing the most suitable method along with the right protocol and the appropriate setup. Understanding the details of each step is quite fundamental to successfully apply these techniques and to adapt them as necessary. To this end, the next section focuses on the specifics of the individual steps of solvent‐based tissue clearing.

## A step‐by‐step guide through high‐throughput DISCO clearing

Regardless of the chosen protocol, solvent‐based clearing includes the following steps: perfusion and fixation, decolorization, decalcification, permeabilization, staining, dehydration, delipidation, and RI matching. After the tissue has been cleared, image acquisition and analysis take place, and this differs based on the samples, protocols, and the specific biological questions asked. Here, we provide a detailed step‐by‐step guide to help scientists achieve optimal results and key recommendations about each step.

### Perfusion and fixation

Tissue fixation is a critical step when preparing tissues for clearing. It terminates any ongoing biochemical reaction and may also increase the treated tissues' mechanical strength or stability (Gage *et al*, 2012). The fixation process and type of fixative depends on the experimental design and can be done either actively or passively.

The active version is used for large tissues such as a whole mouse body or human organs. It involves a pump‐driven way of fixation in which the vasculature of the tissue is used to distribute the fixative to the deeper tissues. The first step is washing out the blood and the most commonly used solution to achieve this is phosphate buffered saline with 1% Heparin (PBS‐H). Heparin in PBS‐H reduces the formation of blood clots and occlusions in vessels, allowing better circulation of liquids (O’Brien, 1960). Since blood clots can form within a couple of seconds, perfusion with PBS‐H should be started right after the animal is deeply anesthetized and restrained in proper position in order to remove as much blood as possible. However, it should be noted that vascular labeling requires alterations in this step. In case, the experiment involves vascular labeling, e.g., by injecting Evans Blue, Wheat Germ Agglutinin, or antibody labeling, using heparin is not recommended as it is likely to wash out the labeling (Todorov *et al*, 2020). There are some tips for evaluating the quality of pump‐driven perfusion. For instance, liver color is a good measure of the quality of perfusion; after adequate perfusion with PBS‐H, the liver color should turn from dark red to pale light brown/yellow.

The second step involves fixing the tissue with a fixative, most commonly paraformaldehyde (PFA) or formalin, using perfusion. In perfusion of rodents, due to the protein cross‐linking that takes place during this fixation muscles can be fired and contractions can be observed in different parts of the body, especially in the tail. These are indicators of adequate perfusion of the fixative throughout the body (Thavarajah *et al*, 2012). On the other hand, strong predictors of an unsuccessful perfusion are extremely inflated lungs and fixative escaping through the nose of the specimen, in which case the perfusion needle is likely in the right ventricle putting pressure on the pulmonary circuit. Repositioning the needle back to the left ventricle may remedy this. If organ‐level clearing is desired, passive fixation is an option but it is possible that the areas deeper in the tissue may potentially remain less transparent in contrast to active perfusion. In any case, over‐fixation must be avoided, as PFA cross‐linking of tissues can obscure antibody binding sites leading to incomplete immunolabeling. We also have observed that over‐fixation causes insufficient transparency and leads to a yellowish color of the tissue after clearing, lowering the overall signal quality in the samples during imaging.

#### Recommendations

1. It is important to prevent the decline of the cardiovascular circulation due to excessive anesthesia, prolonged/repeated trials of inserting, and securing of the perfusion needle, etc. The heart has to contract with at least 1 Hz at the time of starting the perfusion of fixative. We recommend that the same person performs all steps described above until satisfactory tissue‐clearing results are achieved, in order to maximize consistency when handling the samples.
2. The mode of fixation and type of fixative depend on the aim of the experiment. Whole‐body clearing requires perfusion of the fixative for deeper fixation.
3. Heparin in the perfusion solution facilitates the circulation by preventing the cloth formation but it is not a must. Heparin concentration should be adjusted according to the purpose. For example, for vascular labeling heparin in perfusion solution is not recommended to avoid wash out the labeling.
4. The speed of perfusion should closely match the upper limit of the physiological pressure and volume of the heart in order to obtain best results, i.e., the most complete perfusion without bursting capillaries.
5. Over‐fixation should be avoided as it can jeopardize the immunolabeling quality and decrease the level of transparency causing the sample to have a yellowish color.
6. After harvesting the organ(s)/specimen, we suggest keeping the sample at 4°C for optional post‐fixation or until the staining/clearing begins.

### Clearing

#### Decolorization

In optical tissue clearing, light absorption by specific biological substances is a major obstacle. These light absorbing pigments accumulate in tissues and create autofluorescence emission, consequently reducing the tissue signal quality (Tuchin, 2015). For better clearing and deeper imaging, pigments should be eliminated as efficiently as possible. The most prevalent pigments are hemoglobin, melanin, and lipofuscin (Hegyi *et al*, 2011). So far, the decolorization in clearing has mostly focused on the removal of heme groups from hemoglobin. Although perfusion of the animal to wash out the blood is one way to decrease the autofluorescence, it is usually not enough. Remaining heme groups can still hinder imaging especially in organs with high blood content, such as the spleen, bone marrow, and liver. A common approach is either to bleach the heme group or dissociate and wash it out, using peroxides, acid acetones, or strong bases, respectively (Richardson & Lichtman, 2015). The most frequently used reagents for decolorization are SDS or amino alcohols. Quadrol (N,N,N′,N′‐Tetrakis(2‐hydroxypropyl)ethylenediamine) combined with ammonium solutions is another approach for decolorization (Susaki *et al*, 2014). The second light absorbing pigment is melanin, most abundantly present in the skin and the eyes. The removal of the skin and the eyes, in case they are not fundamental to the hypothesis being tested, or using albino transgenic animals will reduce the autofluorescence coming from these sources. Lastly, lipofuscin is an age‐dependent accumulated pigment in the adult brain. One way to avoid lipofuscin is using young animals, but it nevertheless remains an unresolved problem when handling human samples and aged animals (Moreno‐García *et al*, 2018).

##### Recommendations

1. Organic pigments accumulated in organs cause autofluorescence and reduced tissue clearing efficiency. To avoid this, a decolorization step is recommended.
2. Bleaching or dissociation with chemicals is an option for removing heme. However, these procedures are often disruptive to the tissue and can cause damage and bleaching of fluorescent proteins. Amino alcohols are known not to cause damage or alter tissue signal (Susaki *et al*, 2014).
3. To minimize the unfavorable effects of pigments, melanin‐rich organs (skin and eyes) can be removed and/or young animals can be used to avoid lipofuscin.
4. Lipofuscin and melanin remain an open challenge in the decolorization process, and further research is necessary to find optimal ways to overcome them.

#### Decalcification

Bone tissue has a unique composition; soft tissue which forms the bone marrow and the highly calcified hard tissue. When imaging the organs enclosed in bone structures, reducing the scattering of light through the bone matrix is particularly important (Genina *et al*, 2008). A decalcification step is recommended to make bones and bone encapsulated organs (i.e., the brain inside the skull and the lungs inside the ribs) more permeable to clearing chemicals (Verdenius & Alma, 1958). Traditionally, formic acid, nitric acid, trichloroacetic acid, hydrochloric acid (HCl), or chelating reagents such as ethylenediaminetetraacetic acid (EDTA) are used for the decalcification of the bone matrix (Tainaka *et al*, 2014; Cai *et al*, 2019; Pan *et al*, 2019). EDTA can be used for whole‐body decalcification upon perfusing in fixed whole‐body samples (e.g., in PEGASOS and vDISCO methods). It should be noted that the EDTA concentration influences the shrinkage strength, with higher EDTA concentrations resulting in stronger shrinkage. Therefore, it must be adjusted depending on the nature of the sample and the question of interest. Moreover, the shrinkage and distortion of the tissue may differ depending on whether the organ was dissected or the body was intact.

##### Recommendations

1. Bone clearing can be performed either by using high RI solutions (RI = 1.555–1.564) or by subjecting the sample to decalcification with chemicals.
2. EDTA is the most commonly used agent to remove Ca^2+^, and it can be used via active perfusion or passive incubation.
3. EDTA concentration is directly proportional to the amount of shrinkage; the higher the EDTA concentration, the more shrinkage takes place. Thus, depending on the nature of the sample, the concentration should be adjusted.

#### Permeabilization and staining

Permeabilization facilitates the penetration of both clearing chemicals and labeling agents (e.g., dyes and antibodies) and it is therefore fundamental especially for whole‐body clearing and labeling. Tissue permeabilization eliminates several barriers including lipids and extracellular matrix (ECM). In traditional histology, heat or mild detergents such as Triton X‐100, Saponin, or SDS are commonly used to dissolve the lipid bilayer and give successful results for small samples or 1–2 mm sections (Jamur & Oliver, 2010). In iDISCO and derivatives, methanol and methanol/DCM mixtures are used for permeabilization through delipidation (Renier *et al*, 2014). SHANEL on the other hand involves a relatively complex protocol where a methanol/DCM mix is used to delipidate the tissue, acetic acid to hydrolyze the ECM, guanidine + HCl to extract ECM proteoglycans and CHAPS+ N‐Methyl diethanolamine to further permeabilize the sample, aiming at achieving efficient staining of centimeters‐thick human brain tissue (Zhao *et al*, 2020).

Immunohistochemistry (IHC) of whole mouse bodies requires a stronger approach or even the combination of all strategies. The whole‐body labeling and clearing technology vDISCO combine many of the permeabilization and labeling approaches by using a combination of amino alcohol, Triton X‐100, *N*‐acetyl‐l‐hydroxyproline, and methyl‐β‐cyclodextrin (a cholesterol extractor) to permeabilize the specimen. The technique also makes use of a high‐pressure perfusion system to actively push the reagents in all the tissues for an extensive labeling. In addition to combining these reagents, it uses nanobodies for labeling, as they present several advantages. Firstly, nanobody size is one‐tenth of that of an antibody, which allows better penetration in the tissues and consequently better labeling. Secondly, multiple nanobodies can bind to one epitope enabling a higher signal intensity. Finally, nanobodies can be used at near infrared spectra leading to a higher signal‐to‐noise ratio. This is due to the fact that most components that show autofluorescence are generally in shorter wavelengths, and therefore, the near infra‐red wavelengths used with nanobodies allow avoiding overlap with autofluorescence (Cai *et al*, 2019). Nevertheless, the application of nanobodies in IHC is still limited since nanobodies are mostly available only for genetically expressed fluorescent proteins.

Labeling thick tissues requires dyes with a small molecule size, so that they are able to penetrate deep into the specimen. The small molecule dye propidium iodide (PI) can stain deep in both passive and active labeling and provides stable and strong nuclear labeling in all cell types. It is red‐fluorescent and can therefore be used in multichannel imaging, e.g., together with nanobodies conjugated with far‐red fluorophores. The UV spectrum of 4′,6‐Diamidino‐2‐Phenylindole (DAPI) or Hoechst has very limited light penetration that strongly decays in deeper parts of mammalian tissues. For that reason, they are rarely used for whole‐organ staining, although they are well suited for counterstaining tissue slices.

##### Recommendations

1. Permeabilization of large tissues is essential for IHC, since antibodies and high molecular weight dyes can only stain within a few hundred micrometers.
2. Deeper labeling can be achieved either by nanobody labeling or increased antibody incubation time.
3. Small micelle‐forming detergents (e.g., CHAPS) enable better permeabilization and therefore deeper labeling.
4. Before starting the labeling of big tissues (e.g., whole organs), testing the antibodies and nanoboosters using small tissue samples to prototype (i.e., to test their efficiency and optimal concentration) is highly recommended.
5. Using fluorescent probes with a longer wavelength can effectively defer scattering and allow deeper penetration. Therefore, near‐infrared probes are strongly recommended.
6. As an optional step, users may consider making an incision with a sharp object on a less important site on the tissue to enhance the penetration of the soluble agents. This will improve and speed up the process.

#### Tissue clearing

After staining, the samples are ready to be cleared by several rounds of chemical treatment. The different step in this process essentially removes water and lipids from the tissue and homogenizes the RIs. Several approaches can be used for the dehydration step, each with its own advantages and disadvantages. Commonly used dehydrating agents are alcohols including ethanol in FluoClearBABB (Schwarz *et al*, 2015), methanol in iDISCO+ (Renier *et al*, 2016), and tert‐butanol in uDISCO (Pan *et al*, 2016). However, alcohols often result in poor endogenous signal preservation. THF is an alternative for dehydration and it has the same clearing performance as alcohols, but works better at preserving the endogenous signal. THF is commonly used in protocols such as 3DISCO (Ertürk *et al*, 2012a), vDISCO (Cai *et al*, 2019), FDISCO (Qi *et al*, 2019), and sDISCO (Hahn *et al*, 2019). In some of the protocols, an additional step with DCM treatment is performed for further delipidation and an enhanced clearing performance (Seo *et al*, 2016). Lastly for the RI‐matching step, although BABB is the most common solution used in many protocols, it can be replaced by a range of chemicals. For example, ECi (Henning *et al*, 2019) uses Ethyl cinnamate due to its lower toxicity. Alternatively, BABB can be replaced by DBE or a mixture of BABB with other reagents like in the uDISCO protocol (Pan *et al*, 2016) for better fluorescence signal preservation. When choosing the RI‐matching solution, it is important to consider its compatibility with the labeling probes, as well as the toxicity and corrosiveness of its chemical components. These are usually harsh chemicals that can melt the equipment being used and leave irreversible damage and they should therefore be handled with great care.

##### Recommendations

1. The duration of each incubation is highly flexible and depends on the size of the sample and its lipid content. The incubation steps can be prolonged to ensure complete delipidation. However, shortening is not advised.
2. Since the organic solvents used can melt plastic, it is very important to test whether the plastic containers that are to be used are sufficiently resistant. In our experience, polypropylene‐based materials and all glassware withstand BABB well.
3. Due to the toxic and corrosive nature of the many chemicals used in the clearing steps, minding the safety precautions during the use is strongly advised.

#### Imaging cleared samples

Several theoretical and practical considerations need to be made when imaging fixed, transparent tissue samples and taking advantage of the increased optical accessibility. Mainly three imaging approaches are used, partly depending on sample size: wide‐field fluorescence microscopy, point scanning fluorescence microscopy (PSFM), and light sheet fluorescent microscopy (LSFM). Wide‐field, epi‐illuminated fluorescence microscopes can be used for quality checking and imaging purposes if the components meet the excitation and emission criteria of the probes. Wide‐field microscopes allow cost‐effective and rapid imaging of cleared samples using a camera to capture the entire field of view (FOV), using long working distance objectives in a single run. However, these systems usually have basic‐sensitivity cameras and weak excitation efficiency and tend to bleach the fluorescent probes fast due to the wide‐field excitation. This deteriorates the overall image quality of millimeters‐thick samples by capturing excessive out‐of‐focus information. In our experience, these systems are very useful for basic documentation of staining efficacy and imaging supracellular moieties.

PSFM such as conventional confocal or two‐photon microscopy can yield high‐resolution, optically sectioned images given an adequate selection of objectives for cleared samples. However, for cleared samples in mesoscopic scales, PSFM shows two limitations which greatly reduce its feasibility and throughput. The first one is that PSFM scans the excitation focus point by point across the whole field of view (FOV). When the FOV requires more than 10 million pixels, the scanning‐based mechanism makes the acquisition time for a single frame longer than 1s in a conventional confocal microscope, simply extending the process. The second limitation is that mesoscopic imaging requires low‐magnification objectives to achieve adequate throughput. Comparing to high magnification objectives, low‐magnification objectives have longer working distances as well as larger FOV. In principle, long working distance is indispensable for imaging in thick tissues, and a large FOV can effectively increase the throughput. However, low‐magnification objectives mostly have small numerical aperture (N.A.) due to the architecture of modern light microscopy. A smaller N.A. decreases the lateral resolution of the image linearly but the axial resolution quadratically, which renders an anisotropic resolution as well as an extended point spread function in the axial direction. In brief, PSFM is an ideal modality for high‐resolution imaging but only for a small FOV and thinner samples.

Considering the constraints above for imaging a sample in a mesoscopic scale, LSFM emerges as an ideal solution for volumetric imaging of large cleared samples. A typical LSFM has at least two objectives with their optical axes perpendicular to each other. The detection objective is used for forming images on the 2D image sensor, which is usually a scientific complementary metal‐oxide‐semiconductor (CMOS) camera with high sensitivity and adequate cooling. The excitation objective (illumination objective/lens) is used to create a light sheet from a laser beam, where the plane of the light sheet locates on a conjugate plane of the camera sensor. The unique architecture of the LSFM dramatically improves the anisotropic resolution when using low‐magnification, low‐N.A. objectives (Olarte *et al*, 2018). The axial resolution in LSFM is determined by the thickness of the light sheet, which is governed by the optical properties of the excitation path and is not related to the N.A. of the imaging objective. Furthermore, the implementation of the camera as an imaging sensor also greatly enhances the imaging speed of LSFM compared with PSFM. The enhanced imaging speed is substantial for mesoscopic imaging. Theoretically, PSFM has unlimited pixel count whereas the imaging sensor in a camera has a fixed number and size of pixels and thus a fixed physical frame size. The frame size determines the size of the FOV and the number of pixels determines the sampling resolution it can achieve. Therefore, when imaging a sample with a size larger than the FOV, a motorized stage is indispensable in order to relocate the sample and manage to obtain image tiles for the whole sample.

Modern LSFMs for imaging clearing tissues possess two opposing excitation optics. The architecture of the two‐sided illumination aims at compensating the scattering of the light sheet while traveling in a thicker sample from one side. Among the commercially available LSFM, LaVision Ultramicroscope Blaze III (Pan *et al*, 2016; Belle *et al*, 2017; Klingberg *et al*, 2017) offers a larger space as well as a motorized stage with a sufficiently long traveling distance for imaging large samples as whole mice or human organs. An alternative is MesoSPIM, an open‐source project for mesoscale LSFM that is also targeted toward imaging large samples such as a whole mouse (Voigt *et al*, 2019). MesoSPIM uses a digital‐scanning axially swept light sheet to achieve a homogeneous thickness of illumination virtually across the whole FOV and prevents artifacts from optical obstacles in the sample (Keller & Stelzer, 2010; Dean *et al*, 2015). Therefore, MesoSPIM promises a more homogeneous axial resolution as well as fewer artifacts compared with LSFM which uses cylindrical lenses to create light sheets such as the LaVision Ultramicroscope series. However, the horizontal layout of MesoSPIM makes the use of immersion objectives very complicated and is therefore currently not optimal for acquiring images with sub‐micron resolution.

Irrespective of the chosen imaging approach, an adequate selection of objectives is essential to increase the imaging quality. The selection of objectives should be considered in two aspects, the first one being an appropriate magnification which matches its NA to the pixel size to fulfill the Nyquist sampling criterion. Specifically, the optical resolution is determined by the NA of the objective (Keller, 2006) and when two objects are within distances smaller than 0.61 × λ/NA (i.e., Rayleigh criterion), they cannot be identified as two separate objects by the objectives. In order to explicitly sample the optical resolution, the magnified pixel size should also be appropriately evaluated. The Nyquist criterion gives an ideal evaluation for an effective sampling, which states that the sampling frequency should be at least two times higher than the frequency of the sampled signal. To fulfill the Nyquist criterion in microscopic imaging, the magnified pixel size should be two times smaller than the distance from the Rayleigh criterion. In this case, the acquired images can completely reveal the resolving powers of the microscope. The magnified pixel size is the division of the physical pixel size to the magnification, of which the physical pixel size can be found on the specification sheet from the camera supplier. The second aspect to consider is that special designs can correct the aberration from embedded media. Cleared samples are usually immersed in their RI‐matching medium for microscopic imaging which will cause considerable spherical aberration if the objectives are not chosen correctly. Using an objective which corrects for aberrations caused by the RI‐matching medium is recommended. Clearing methods based on organic solvents typically use a high‐RI solution such as BABB or DBE and need a special objective design that can correct for aberrations in such a high NA liquid. Despite the overall limited number of tissue‐clearing‐specialized objectives, the repertoire of such options has been growing in the recent years. In addition to correcting the aberration, an immersion objective or even a dipping objective with a dipping cap can prevent recording perturbations that take place at the liquid surface of the medium in the chamber. Image acquisition in large‐scale imaging usually takes from several hours up to several days and environmental disturbances such as air flow or people’s movements in the room usually cannot be avoided at such a time scale. In fact, imaging a sample in an organic‐solvent‐based RI‐matching medium needs to be performed in a room with sufficient ventilation, which will cause a strong air turbulence and sometimes might vibrate the liquid surface of the medium. The effect of these will be detected by the camera. Immersion objectives eliminate the gap between the medium and the objectives, thereby circumventing the possibility of such perturbations.

##### Recommendations

1. Choose adequate microscopy modalities according to the desired spatiotemporal resolution and FOV. For daily quality checking and documentation, we suggest using epifluorescence microscopes. These can also be used when trying to decide whether it is preferable to continue with a high‐resolution PSFM or LSFM scans. High magnification and high NA imaging in a small FOV are better suited for PSFM than LSFM. For large FOV and volumetric imaging with moderate magnification and NA, LSFM is the best choice.
2. The imaging medium must be optically matched (RI for given wavelength bands) and chemically compatible with the imaging objectives to ensure the best possible image quality.
3. Sample size is a major determinant when deciding for imaging modality. Imaging whole organs usually requires LSFM, but PSFM or epifluorescent systems may work with limited quality. Before imaging a large sample such as a whole mouse body, the size of the chamber and the moving distance of the motorized stages needs to be carefully checked. The size of the imaging chamber should be ideally at least 2 times larger than that of the sample (in all dimensions).

#### Data handling and analysis

The last and perhaps the most important steps are to visualize, analyze, and interpret the data. These steps are under intense investigation and undergo constant improvement. The computational demand continuously increases due to the increasing complexity of imaging data analyses. Usually, the scans comprise a huge amount of high‐bit‐depth (traditionally 8, 12, or 16 bit) images that may easily take up terabytes of disk space for one specimen, making storage very demanding for conventional data storage strategies. Compressing the data allows substantial time saving when transferring the data between distant storage and computational units. Many lossless image compression algorithms exist for storing scientific imagery. However, compressed images can only be read with the matching format decoder. Researchers often face the need to transfer their data into different software for subsequent processing or visualization, but surprisingly only a handful of compressed image formats are universally available to different software. One could convert between the different image formats, but this is time and resource inefficient as well as prone to conversion errors. Hence, such conversion procedures should ideally be kept at a minimum. We based our data storage on the Lempel‐Ziv‐Welch (LZW) lossless compression, which to our experience is the most compatible across the various image analyzing and rendering software. Fortunately, a growing number of advanced scientific‐grade data storage options (to store metadata besides plain imagery) became available such as the Hierarchical Data Formats (e.g., HDF5), the KLB format (Amat *et al*, 2015), the B^3^D format (preprint: Balázs *et al*, 2017) which will hopefully eventually be implemented in all widely used research software.

The need for compression is due to the reasons mentioned above, one being the large amounts of data generated. The generation of large amounts of data partially stems from imaging large samples that exceed a single FOV of the microscope. This can be resolved by scanning multiple FOVs, also referred as *tiled* or *mosaic* scans. These tiles most often need to be precisely aligned and fused to reconstruct the whole scan for subsequent quality control and processing. We highly encourage users to acquire spatially overlapping tiles (practically ca. 8‐20% at least) to prevent unrecoverable signal loss at the stitching borders (also known as seams) and to target a homogenously illuminated dataset at the end. Tiles are either produced by the motorized stage of the microscopes or by manual repositioning of the sample after each acquisition. Thus, there are different stitching procedures based on the acquisition strategy and the desired precision of alignment. Generally, we distinguish manual, semi‐ and fully automated image stitching methods. One option is the epifluorescent AxioZoom (Zeiss), which can be used even without a motorized sample stage and captures 2D tiles that can be manually aligned and fused along the XY‐plane with Photoshop via the “Photomerge” function (Herrmann *et al*, 2018). However, this requires that the user sits in front of the computer during the entire procedure. Although this setup allows scanning 3D stacks, the objective only provides a very low depth of view focus and is therefore rarely used for volumetric scans of cleared tissue. Another option for improving the contrast or image quality by deconvolution, when using confocal microscopy, is the Zen Black (Zeiss) software that can automatically align the tiles into a fused stack using the “online stitching” function. Multiple stacks can be further stitched using several methods. For example, Fiji (Schindelin *et al*, 2015), an open‐source distribution of ImageJ, offers 10 stitching plug‐ins. Some are considered as semi‐automated stitching methods (such as the *Stitch Sequence of Grids of Images*) and are in essence repetitive procedures, which require the user to input the correct displacement offsets in X and Y directions of the tiles and then applies the same offset to all the z‐slices, by assuming that there is no displacement in the Z direction. Additionally, intensity cross‐correlation algorithms (in the aforementioned Fiji plugin it is called *compute overlap*) can be used to find the XY displacement offsets and then the stitching can be left to finish without further user intervention. After stitching with Fiji, we suggest an additional LZW compression step as the current licensing prohibits writing LZW TIFFs (Tagged Image File Format) natively. Furthermore, the state‐of‐the‐art 3D stitching tools enable fully automated and parallelized alignment and fusion of Terabyte sized volumetric tile scans even with single‐axis‐rotation (e.g., static camera rotation) and z‐shifts across the tiled volumes. The major noncommercial solutions are TeraStitcher (Bria & Iannello, 2012) and BigStitcher (Hörl *et al*, 2019) which run on Linux, Mac, and Windows operating systems. To our experience, TeraStitcher is a good choice for high‐resolution brain vasculature images, where the continuity of the fluorescent signal of the curvilinear shapes across multiple tiles is critical. The advantage of these stitchers is that after the initial setup, the computer automatically finds the displacement offset pairs and finally reconstructs the entire 3D scan optionally in multiple resolution levels. Fortunately, both alternatives are capable of parallel graphics‐processing‐unit‐based acceleration of displacement finding (preprint: Balázs *et al*, 2017) and automatic compression of the reconstructed volumes, which significantly speeds up the entire process. BigStitcher is available as a Fiji plugin and offers additional functionalities besides stitching (including multi‐angle stitching, deconvolution, and illumination selection) to compensate for optical effects. However, it currently suffers from the RAM handling problems of Fiji (Bria *et al*, 2019). Recently, proprietary software like the Tile Sorter plugin of Vision4D (Arivis) and Imaris Stitcher (Bitplane) became available and offer convenience to scientists working in these integrated platforms. Vision4D provides a critical niche for 3D reconstruction by manual setup (with landmark coordinates in the overlapping scans to define the final plane of stitching consecutive scans) which allows rigid transformation and optionally scaling to match the joined volumes as good as possible.

Following sample reconstruction, visual inspection and quality control are performed, and they require tools for viewing and rendering the scans. Nowadays, all major viewing platforms provide a growing number of data analysis functions, from tracking and geometry reconstruction to statistical quantification. One of the most frequent free programs is Fiji that works with most voxel‐based imaging data formats and has extensive community‐based support. This has made Fiji a popular choice for novice users and for sharing among collaborators. However, it is relatively limited in 3D rendering and we therefore recommend it as a tool for viewing cleared tissue datasets especially in combination with the virtual stack feature. The BigDataViewer (Pietzsch *et al*, 2015)—available as a Fiji plugin—is specifically developed for investigating scans of cleared tissue in an attempt to overcome this limitation. The most popular high‐performance 3D image reconstruction software for fluorescent and electron microscopy‐based biomedical research are Imaris (Bitplane), Vision4D (Arivis), and Amira (Thermo Fisher Scientific). All three programs provide built‐in downstream analyses such as object counts, distance measurements, or shape tracings and their combination even in their built‐in scripting environment. They also allow high‐quality 3D renderings on adequately high‐end workstations and clusters. Notably, the recent Imaris Viewer enables state‐of‐the‐art volumetric rendering as a free package. Additionally, Vaa3D (Peng *et al*, 2010), ClearMap (Renier *et al*, 2016), Neurolucida (Dickstein *et al*, 2016), NeuroGPS Tree (Quan *et al*, 2016), and CUBIC Atlas (Murakami *et al*, 2018) are powerful alternatives that target further downstream analysis specifically developed for tissue‐clearing data. Since the range of functions differ very significantly, we suggest establishing and evaluating multiple programs in the laboratory. Recently, machine learning techniques for image analysis also gained popularity as a step toward unbiased and high‐throughput analysis of multi‐terabyte sized imaging data. Some examples are Deep learning‐enabled Metastasis Analysis in Cleared Tissue (DeepMACT) (Pan *et al*, 2019), for quantification of single‐cell cancer metastases, SHANEL (Zhao *et al*, 2020), a pipeline to statistically quantify millions of cells in cleared human brain tissues and Vessel Segmentation & Analysis Pipeline (VesSAP) (Todorov *et al*, 2020) which quantifies the vasculature across the different regions of the mouse brain. Commercially available platforms (Imaris, Vision4D and Neurolucida) have also started to incorporate machine learning algorithms for image analysis, which highlights the growing need of scientists to reliably quantify big data without human intervention.

##### Recommendations

1. Data compression after image acquisition provides advantages in terms of time, storage, and transfer.
2. Stitching can be done in several ways depending on acquisition. Photoshop, Fiji (ImageJ), TeraStitcher, and BigStitcher are common noncommercial available options.
3. Imaris and Vision4D allow the best quality 3D rendering, initial image analysis, and video options in a single proprietary package.
4. Further analysis can be done using software from research publications. Vaa3D, Neurolucida, CUBIC Atlas, ClearMap, NeuroGPS Tree, or deep learning algorithms can be used depending on the nature of the analysis such as DeepMACT, SHANEL, and VesSAP.

## Current main challenges on DISCO clearing based on survey results

In order to get a more informed overview of the challenges current users have faced when utilizing solvent‐based clearing methods, we conducted a survey via phone interviews and an online questionnaire. We reached out to tissue‐clearing users from the following sources: scientists from our former workshops, some authors of the publications that utilized tissue clearing and through social media. The participants provided information related to their field of research, the clearing protocol they utilize, the type of tissue they clear, the challenges they face or have faced and also shared anticipated advancements in the field. As the shared challenges were not necessarily specific to a particular clearing method, we tried to address them in a general manner in order to aid novice users of tissue clearing and to help users troubleshooting.

The majority of the users mentioned the problem of “losing endogenous GFP signal”. We know that techniques using ethanol for dehydration, such as 3DISCO, can cause quick quenching of the signal, limiting the applicability of the technique. To overcome this bottleneck and prolong the preservation time of the signal, we can recommend uDISCO due to the 4:1 DPE content of the final RI‐matching solution and the addition of vitamin E which prevents reduction reactions in the solutions. Additionally, we often use vDISCO, as nanobodies boost the fluorescence signal ~ 100 fold and also retain the signal for over a year. A wide range of nanobodies are available now, and in case they are available for the user’s applications of interest we would highly recommend using them. Additionally, it is now known that the pH of the solutions used is very important for retention of the GFP signal. fDISCO utilizes high pH which also helps with preserving the signal longer. The choice of chemicals might also affect signal preservation. For example, the lack of oxidation inhibitors in the reagents from some brands leads fluorescence loss. To avoid this, using products that include oxidation inhibitors is preferable. Alternatively, inhibitors or vitamin E can be manually added to the reagents immediately after opening the bottles and before starting the clearing procedure. Finally, one could also test water‐based clearing solutions such as PACT and CUBIC.

Another issue brought up in the survey was the “presence of autofluorescence and background”. We are aware that it is challenging to reliably image and quantify signals from endogenously expressed fluorescent proteins in large cleared organs and whole mouse bodies due to the low signal contrast. The skeletal muscle and other bodily tissues have obstructive autofluorescence in the visible spectrum and therefore interfere with fluorescent proteins such as EGFP, EYFP, and mCherry which are commonly used in transgenic animals and emit light in this range. In addition, fluorescent proteins are often less bright compared with most synthetic fluorophores and their signal intensity is further attenuated during the clearing and imaging procedures. For these reasons, we prefer using vDISCO, which involves an immunolabeling technology based on multiple fluorophore labelled nanobodies to enhance the signal of fluorescent proteins by up to two orders of magnitude. Nanoboosters can also be used to replace the emission of the of the endogenous signal from visible light to far red (640–700 nm) or far‐far red (700–800 nm) spectra which are more distinguishable from the autofluorescence emission (between 270 and 490 nm, broadly around 580 nm for residual blood and 690 nm for age‐related neuropigments). If vDISCO is not a suitable option, we would suggest performing a CuSO_4_ treatment to reduce the autofluorescence. The concentration is a critical determinant in this step as too high CuSO_4_ concentrations can cause the sample to become blue leading to more autofluorescence. Additionally, the pretreatment steps can be switched for optimizing the quality as was shown in the PEGASOS procedure by Jing *et al* (2018). Another option for increasing the signal‐to‐background ratio during imaging is narrowing the bandwidth of the emission filter and centering its spectrum at the peak of the emission. This improves the ratio because the broad‐band filter simply collects more background fluorescence.

The third challenge that was reported was the “complexity of the procedure and immunolabeling”. The advantages of solvent‐based clearing techniques are their ease of applicability as they require subsequent solvent immersion over a period of time. The protocols are straightforward because they are based on the sequential immersion of the tissue of interest in organic solvents and simply consist of pretreatment, labeling, and clearing steps. However, it is true that immunolabeling is an ever‐evolving topic in the field. While antibody labeling is used because of its wide range of applicability, it is also known to have specificity problems and usually only shows moderate penetration due to its size. Small molecule dyes are preferred because of their deeper penetration, but unfortunately not every epitope has a specific dye. Current efforts aim at improving the available labeling options. One recent example is the vDISCO technology that utilizes pressure‐driven administration of nanobodies to address the disadvantages.

Along the same lines, several users reported “difficulty in clearing clinical samples and overfixed big tissues”. Poor antibody penetration and antibody compatibility issues were also mentioned. Generally, our suggestion for overcoming the poor penetration issue is using the SHANEL protocol, which has shown to work on aged human samples and very large tissues (i.e., whole human brain, kidney, eye, whole pig brain). CHAPS, the detergent used in this protocol, allows better penetration by forming tiny micelles that stretch the tissue to generate space for deeper labeling and improved imaging quality. However, it should be noted that this procedure is rather time‐consuming compared with others. Regarding antibody compatibility, we would highly recommend testing antibodies in small samples before applying them to bigger samples or when using different protocols. To our knowledge, there is no study that correlated the stability of different types of antibodies for labeling and maintenance.

The “time required for the procedure” was also brought up as a challenge. The time required for a procedure would highly depend on the size and the pigmentation of the specimen. Additionally, the protocols can be individually optimized depending on the nature of the question addressed. For example, if the tissue is composed of bone material, it may be required to prolong the decalcification step, while if the tissue is small and soft this step can be minimized. The same applies to delipidation or dehydration; for smaller samples, the time in each step can be reduced. It should be nevertheless noted that some techniques, such as vDISCO, require longer time irrespective of the sample size. The pretreatment is a step that can be shortened or eliminated completely depending on whether it is required due to the nature of the sample. In all cases, when deviating from the published protocols, we highly recommend testing the effect of the changes on non‐critical but representative samples.

Generally, tissue shrinkage could represent an advantage or a disadvantage. One of the biggest disadvantages brought to our attention was the “shrinkage of specimens which might alter the anatomy of the tissue”. There are several steps that will cause specimen shrinkage and adjusting the steps according to the desired outcome will result in an undistorted tissue. The first step that will cause shrinkage is decalcification. The EDTA concentration influences the amount of shrinkage and if the specimen is not dissected it is possible that different tissues will shrink differently causing distortion. The second step that effects shrinkage is permeabilization, which disrupts the extracellular matrix to allow deeper access to the molecular probes while at the same time causing anisotropic shrinkage. If keeping the microscopic structures intact is fundamentally necessary, we suggest using a protocol that does not have a permeabilization step such as 3DISCO or uDISCO. The third step that influences shrinkage is sample dehydration. This step is highly dependent on the tissue type, and the higher the water content in an organ the more shrinkage is caused in the tissue. For example, bones are stiff and rigid but the spleen is comparably softer and has a higher water content. To minimize the effect of differences in tissue composition, the number of steps during the dehydration process could be increased allowing a smoother process. Another important aspect to consider for dehydration is the type of the dehydrating agent. As an example, iDISCO utilizes alcohols which are known to distort cell nuclei, explaining the anisotropic shrinkage.

For some, “reagent toxicity” is an issue. Therefore, we decided to share a table with the safety levels of most solvent‐based clearing reagents and some additional information (Table 2). For further details, open databases such as PubChem, MSDSonline provide an elaborate sheet on how such solutions are to be handled. Gloves, laboratory coat, and masks are necessary during exposure to these reagents. The disposal of these reagents should also be performed according to the relevant safety regulations.

**Table 2:** Toxicity of the most commonly used chemicals in solvent‐based clearing.

Name of the chemical	Method	Safety level	Information
Benzyl Alcohol Benzyl Benzoate	BABB, FluorClearBABB, uDISCO, vDISCO, fDISCO, sDISCO, SHANEL	Mild precaution required	Both chemicals are commonly used in cosmetics
Tetrahydroflurane (THF)	3DISCO, iDISCO, vDISCO, sDISCO	Moderate precaution required	Avoid inhalation of the vapor, might cause nausea, dizziness, headache…
Dibenzyl Ether (DBE)	DBE, eDISCO, fDISCO, sDISCO	Mild precaution required	Commonly used food additive, no harmful effect
Ethyl Cinnamate (ECi)	ECi, BALANCE, EyeCi	Safe	Ethyl cinnamate is an FDA‐approved food flavor and additive to cosmetic products
Tertiary Butanol	uDISCO, PEGASOS	Moderate precaution required	Vapor is narcotic and irritating in action. Liquid is irritating to skin and eyes
Dichloromethane (DCM)	3DISCO, iDISCO uDISCO, vDISCO, SHANEL	Moderate precaution required	As an artificial compound, it irritates the skin and eyes
N,N,N′,N′‐Tetrakis(2‐hydroxypropyl) ethylenediamine (Quadrol)	vDISCO, PEGASOS	Severe precaution required	Quadrol can cause skin irritation, serious eye damage and serious eye irritation
Ethylenediaminetetraacetic acid (EDTA)	vDISCO, PEGASOS	Safe	EDTA is a safe compound to use; it only irritates the eyes, skin, and respiratory tract in high exposures
Methanol	iDISCO, SHANEL	Moderate precaution required	High concentrations can produce central nervous system depression and optic nerve damage. Can be absorbed through the skin

“Equipment availability” came up in our survey as a challenge. However, we would like to mention that all material used in the protocols mentioned above are available online and commercialized. Additionally, new LSFM core facilities can be contacted for collaborations. To lower the costs, some sample holders may also be 3D‐printed at the desired size. Furthermore, relatively smaller samples could readily be imaged by more widely available confocal and 2‐photon microscopes. For the vDISCO active clearing pipeline, the pumps can be used for up to 4‐6 animals, minimizing the required number of pumps upon synchronized experiments.

While the “lack of automated quantification techniques” for different organs and clearing methods indeed represents a challenge, we believe that the field is progressing toward generating more resources to allow automated, reliable quantification. A broad explanation was given in the data analysis section but to exemplify; Clearmap, VesSAP, DeepMACT, NeuroGPS Tree, Neurolucida, Imaris, Vision4D, and ImageJ/Fiji are common predefined and reviewed solutions that could provide tremendous help during analyzing data until problem‐specific custom code is (if really needed) implemented in‐house.

Finally, Z‐resolution depends on several factors and the challenge of the “Z‐resolution being generally lower than the x and y resolution” can be partially controlled by thinning the light sheet which is usually termed as the NA. Alternatively, new LSFM utilizing axially swept light sheet such as MesoSPIM are promising for obtaining a more homogenous light penetration through the tissue, partially addressing this shortcoming. So far, LSFM is a growing field in microscopy and novel designs such as ctASLM (Chakraborty *et al*, 2019) or oblique plane microscopy (Sapoznik *et al*, 2020) substantially improve the resolution in xy and z, making the overall resolution better and more homogeneous. However, these new modalities are not commercially available, and it is therefore required that experts establish and maintain the microscope locally.

## Future perspectives

Tissue clearing revolutionized classical histology and expanded the amount of information that can be extracted from large intact tissues. Given the advancements in this field during the last decade and ongoing improvements, there can be little doubt that tissue clearing technologies will continue to evolve in the near future. We foresee that new protocols will continue to further improve labeling efficiencies and imaging qualities but more importantly will provide more robust, unbiased quantification methods using deep learning algorithms. Faster and more accurate pipelines for stitching and organ segmentation as well as ways to handle large datasets are urgently needed. Despite the open challenges, the combination of different fields: tissue clearing, labeling, imaging, and recently deep learning has provided significant improvements so far. These improvements have provided a system‐level understanding in a variety of contexts, ranging from fine cellular details, to the discovery of new anatomical structures to macroscopic level whole‐body analyses of single‐cell metastasis.

The next big advancement in the field perhaps could be linking complex biological function to tissue structures, something that has remained challenging, due to the need to work with fixed samples. This advancement could be integrated with omics analyses, e.g., transcriptomics, metabolomics, or proteomics which would lead to an unbiased understanding of an intact specimen not only structurally but also functionally. In turn, these techniques can be applied to generate organ maps, monitor disease progression, and test treatment options. Along the same lines, drug development can greatly benefit from the type of analyses that can be performed using tissue clearing.

The increasing interest in studying biological processes in thick samples will likely spur these and other improvements in the future and it may become even harder to decide which would be the optimal protocol to pursue for a specific scientific question. In this tutorial, our intention was to share our knowledge, experience, and best “tricks” to help scientists who are currently trying to establish these techniques. We have addressed several common struggles to reach higher level of transparency, and deeper labeling, signs of proper tissue processing and preservation of fluorescent signal. Furthermore, we provided an overview on the toxicity of reagents and on the applications of tissue clearing as examples to find relevant protocols faster.

### Author contributions

All aspect of the manuscript: MM and ZIK; Literature review, revision and improvement in the manuscript: MIT; Revision of the optics and physics parts of the manuscript: T‐LO; Supervision of the work: AE; Reading and final approval of the manuscript: All authors.

### Conflict of interest

The authors declare that they have no conflict of interest.
